# A method to rapidly create protein aggregates in living cells

**DOI:** 10.1038/ncomms11689

**Published:** 2016-05-27

**Authors:** Yusuke Miyazaki, Kota Mizumoto, Gautam Dey, Takamasa Kudo, John Perrino, Ling-chun Chen, Tobias Meyer, Thomas J. Wandless

**Affiliations:** 1 Department of Chemical & Systems Biology Stanford University, Stanford, California 94305, USA; 2 Department of Biology Stanford University, Stanford, California 94305, USA; 3 Cell Sciences Imaging Facility Stanford University, Stanford, California 94305, USA

## Abstract

The accumulation of protein aggregates is a common pathological hallmark of many neurodegenerative diseases. However, we do not fully understand how aggregates are formed or the complex network of chaperones, proteasomes and other regulatory factors involved in their clearance. Here, we report a chemically controllable fluorescent protein that enables us to rapidly produce small aggregates inside living cells on the order of seconds, as well as monitor the movement and coalescence of individual aggregates into larger structures. This method can be applied to diverse experimental systems, including live animals, and may prove valuable for understanding cellular responses and diseases associated with protein aggregates.

## Abstract (web summary)

Protein aggregates are associated with a wide variety of diseases. Here, in order to address how protein aggregation affects cellular homoeostasis, the authors describe a method to rapidly create protein aggregates in living cells and organisms with precise spatial and temporal control.

## Graphical abstract

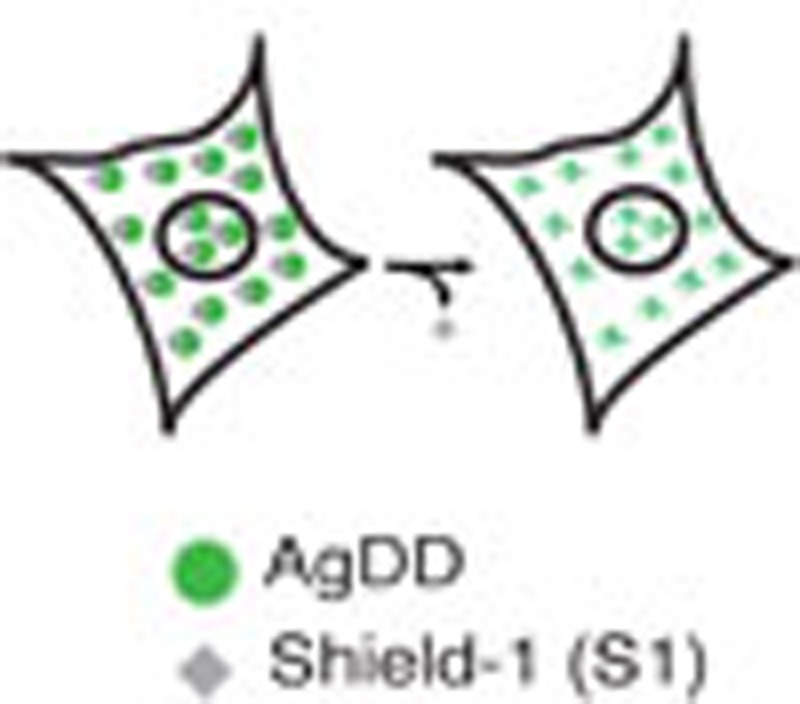

Proteins are involved in every aspect of cellular function and homoeostasis, playing enzymatic, structural or signalling roles that are typically made possible when the polypeptide folds into a particular three-dimensional conformation. However, proteins do not always achieve their folded conformations during protein synthesis, and are folded; mature proteins may be readily damaged by various types of environmental stresses such as heat and oxidative stress. Misfolded proteins often lose the ability to perform their intended functions, and in some cases misfolded proteins can acquire new pathological functions. Thus, cells have developed a variety of mechanisms to deal with misfolded proteins and utilize a significant amount of energy to maintain cellular protein homoeostasis^1^.

Cells attempt to cope with misfolded proteins using two major pathways: the protein refolding and degradation^2^,^3^. Deficient protein homoeostasis is strongly associated with neurodegenerative diseases including Alzheimer's disease, Huntington's disease, amyotrophic lateral sclerosis and Parkinson's disease^1^,^4^. It is widely recognized that protein aggregation is often associated with neurodegenerative diseases, and is also strongly associated with aging. For these reasons, one potential therapeutic strategy for treating neurodegenerative and other age-related diseases is to prevent the formation of protein aggregates by modulating protein homoeostasis^4^,^5^.

Although protein aggregates are associated with a wide variety of diseases, we do not yet understand the mechanisms underlying protein aggregation, including how cellular aggregates are formed, how they are cleared, how other cellular proteins are involved in aggregate formation and clearance or why oligomers and aggregates are toxic to cells^6^. Many studies have used forceful perturbations, such as heat shock and proteasome inhibition to generate aggregates^7^,^8^. However, these stressors are extremely strong and nonspecific, concomitantly inducing global cellular stress responses unrelated to protein aggregation^9^. Others have studied aggregates using genetic perturbations including polyQ-fused mutant Huntingtin (Htt) proteins^10^,^11^. Despite higher specificity, these systems display relatively little control over the timing of aggregate onset or extent of aggregate formation.

In this study, we describe a novel small molecule-dependent engineered protein that can be used to rapidly create protein aggregates in cells with a high degree of spatial and temporal control. The ability to regulate the formation of aggregates should enable advances in our understanding of how protein aggregation affects protein homoeostasis in cells.

## Results

### Development of chemically controllable protein aggregates

To develop a conditional system to generate protein aggregates in cells, we utilized destabilizing domain (DD) technology developed by our group^12^,^13^,^14^. This technology was first developed as a method to regulate the metabolic stability of any protein of interest using a cell-permeable ligand in conjunction with a genetically encoded DD domain. We found that these DDs confer instability to any fused-partner proteins and cause proteasome-mediated degradation of the entire fusion protein^12^,^13^,^14^. High-affinity ligands bind to and stabilize these DD-fused proteins in a dose-dependent manner^12^,^13^,^14^. Previous biophysical studies showed that DDs possess a interesting property; the folding state of the DD can be reversibly regulated by the presence or absence of the ligand^15^. We took advantage of this behaviour to interrogate the cellular protein quality-control surveillance mechanisms using DDs as model substrates for unstable proteins^16^,^17^.

**Figure 1:**
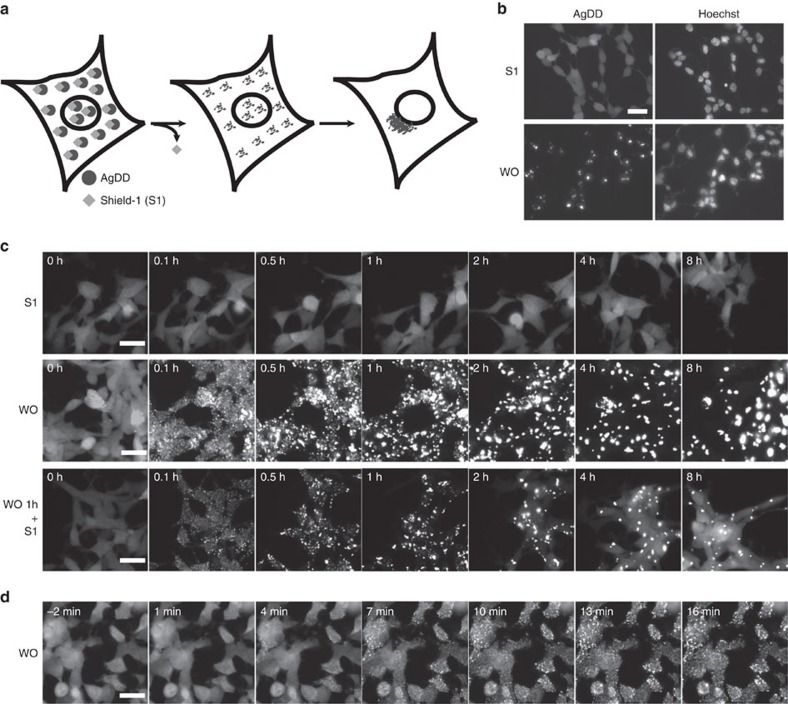
Development of AgDD to rapidly create protein aggregates in living cells. (**a**) Schematic representation of inducing aggregated proteins inside cells using an AgDD. (**b**) Images of representative HEK cells stably expressing AgDD before and after S1 washout (WO) for 150 min. Scale bar, 10 μm. (**c**) Time-lapse images of representative HEK cells stably expressing AgDD. Top: cells cultured in media with S1 throughout the experiment; middle: cells cultured in media with S1 withdrawn at 0 h and bottom: cells cultured in media with S1 withdrawn from 0–1 h then readministered from 1–8 h. Scale bars, 20 μm. (**d**) Early time-lapse images of representative HEK cells stably expressing AgDD following S1 withdrawal. Scale bar, 20 μm.

To develop a new tool to study the mechanisms of protein aggregation and its effects on cells, we engineered new DDs derived from the human FK506 binding protein 12 (FKBP12) protein (Fig. 1a). The hydrophobicity of N-terminal nuclear export signals (NES) has been previously shown to be a critical factor for aggregation^18^. Thus, we constructed several variations of fluorescently tagged DDs with NES sequences fused to their N termini (Supplementary Fig. 1a). These NES-tagged DDs were stably expressed in HEK293 cells, and we observed that one of these DDs, which we call aggregating destabilizing domain (AgDD), aggregates when the stabilizing ligand (Shield-1 or S1) is withdrawn from the cell-culture media.

Beginning with the N terminus, the AgDD comprised of a 10-amino-acid NES sequence (LALKLAGLDI) derived from a cAMP-dependent protein kinase inhibitor, an FKBP-derived DD (L106P) followed by superfolder green fluorescent protein (GFP). When cells expressing AgDD are cultured in medium containing S1, the specific ligand for FKBP-based DDs, AgDD is distributed uniformly in the cells. However, after removing S1 from the culture medium for 150 min, AgDD coalesced into aggregates in cells (Fig. 1b). This phenomenon of aggregation was also observed in other cell lines, including NIH3T3 and U2OS cells (Supplementary Fig. 1b,c).

To further characterize the time-dependent nature of AgDD aggregation, we collected cells expressing high levels of AgDD by flow cytometry and monitored the behaviour of the AgDD protein using epifluorescence time-lapse microscopy. When cells are cultured with S1, GFP is uniformly distributed throughout these cells with no visible aggregates (Fig. 1c and Supplementary Movie 1). When S1 is withdrawn from the culture media, AgDD rapidly unfolds and forms small aggregates (Fig. 1c and Supplementary Movie 2). On closer examination, the formation of small, mobile aggregates commences within minutes of ligand withdrawal (Fig. 1d and Supplementary Movie 3), and the small aggregates coalesce into larger immobile aggregates over time. Quantification of the aggregates reveals that the cellular area occupied by single aggregates increases in time-dependent manner (Supplementary Fig. 1d), and the total number of aggregates decreases once aggregates appeared (Supplementary Fig. 1e).

Aggregates were allowed to form for 1 h and then S1 was re-introduced into the culture media, leading to the re-appearance of diffuse GFP signal by the 2-h time point (Fig. 1c and Supplementary Movie 4). The addition of cycloheximide along with S1 at the 1-h time point did not prevent or significantly attenuate the growth the diffuse GFP signal (Supplementary Fig. 1f), suggesting that newly synthesized AgDD-GFP is not the primary source of the diffuse GFP signal. These results demonstrate that AgDD is a chemically controllable protein that enables the rapid and pervasive induction of protein aggregation with high-temporal resolution. Another advantage of using the FKBP-based DD is that the S1 ligand does not induce global stress responses produced by stimuli like proteasome inhibitors or heat shock^17^,^19^.

We next monitored the aggregation of AgDD using analytical flow cytometry by collecting AgDD-expressing HEK293 cells at different time points after S1 removal, and subjecting these samples to analysis using the PulSA method^20^. Cells with aggregates displayed narrowly distributed fluorescent signals compared with cells that did not have these punctate structures (Supplementary Fig. 2a). Consistent with the time-lapse microscopy experiments, we found that the population of cells that contained aggregates continuously increased following withdrawal of the ligand (Supplementary Fig. 2b).

### AgDD aggregates resemble known aggregates

**Figure 2:**
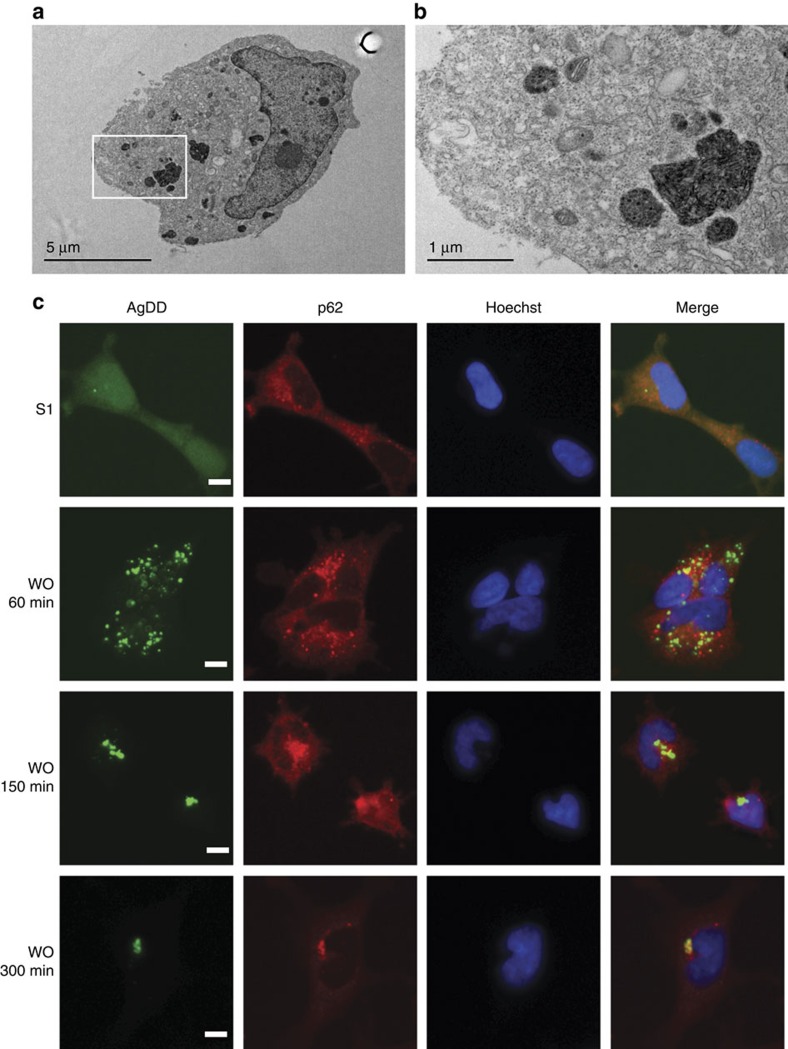
AgDD aggregates have similar characteristics as filamentous aggregates. (**a**) TEM image of representative HEK cells stably expressing AgDD after S1 washout for 60 min. Scale bar, 5 μm. (**b**) Magnified TEM image of white box inset in **a**. Scale bar, 1 μm. (**c**) Immunofluorescence images of representative cells stably expressing AgDD-GFP before and after S1 removal for the indicated times. Left: AgDD; middle-left: p62; middle-right: Hoechst; right: merge. Scale bars, 10 μm. Texas-Red was used to label the p62 antibody.

To further examine the structure of aggregates, we used transmission electron microscopy (TEM) to visualize negatively stained cells expressing the AgDD-GFP fusion protein. Consistent with the results from fluorescent microscopy, when S1 is withdrawn from the culture media, AgDD forms large, electron-dense aggregates (Fig. 2a,b; Supplementary Fig. 2c,d). The ultrastructural characteristics of the aggregated AgDD-GFP resemble known filamentous aggregated proteins, such as the ΔF508 mutant of cystic fibrosis transmembrane conductance regulator (CFTR)^21^.

We also monitored localization of the endogenous p62/SQSTM protein, a well-characterized marker for selective autophagy that is known to colocalize with aggregates^3^. Immunofluorescence staining of AgDD and p62 showed that p62 appears to colocalize with AgDD aggregates, although we observe colocalization only after AgDD has been allowed to aggregate for some time only at later times (5 h following S1 withdrawal, Fig. 2c).

### AgDD aggregates affect cellular fitness

**Figure 3:**
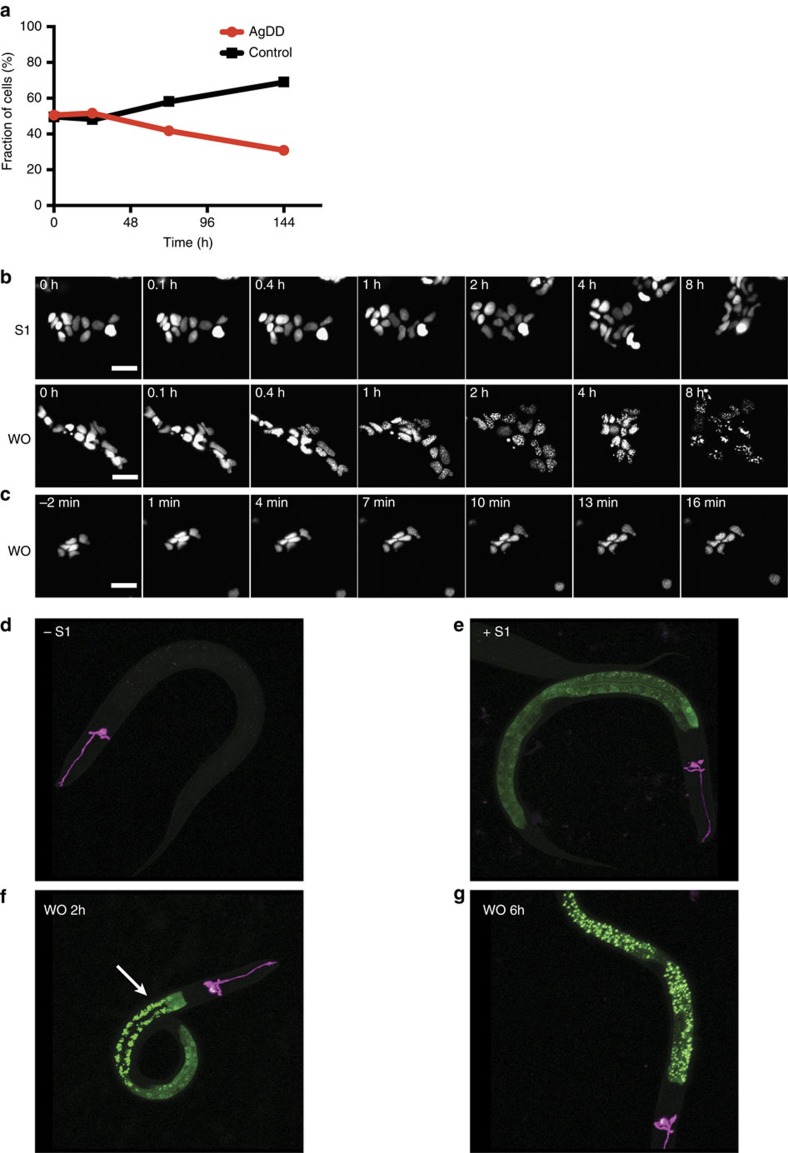
Applications of AgDD. (**a**) HEK293 cells expressing AgDD and HEK293 cells expressing mCherry were mixed and co-cultured in media containing S1. S1 was withdrawn for the indicated times, and cell populations were quantified using flow cytometry. Replicates of *n*=3. Error bar is Standard Deviation (STD). (**b**) Time-lapse images of representative HEK293 cells stably expressing Nuclear AgDD. Top: cells cultured in media with S1 throughout the experiment; bottom: cells cultured in media with S1 withdrawn at 0 h. Scale bars, 5 μm. (**c**) Early time-lapse images of representative HEK293 cells stably expressing nuclear AgDD following S1 removal. Scale bar, 5 μm. (**d**–**g**) Images of representative nematodes expressing AgDD in intestinal cells. Top left: no S1; top right: 50 μM S1, bottom left: S1 removed for 2 h, bottom right: S1 removed for 6 h. Scale bar, 50 μm.

Next, we examined the effects of aggregated AgDD on cell growth and viability. For this purpose, we devised a co-culture proliferation experiment in which HEK293 cells expressing AgDD were co-cultured with control HEK293 cells expressing only mCherry in media containing S1. We withdrew S1 to induce AgDD aggregation and analyzed the number of AgDD-positive cells relative to control cells using flow cytometry. Cell expressing the aggregated AgDD proliferated more slowly than control cells but only after a full day of aggregate induction (Fig. 3a and Supplementary Fig. 3a), similar to results from experiments using polyQ aggregates^22^.

### Aggregates formation depends on the amount of AgDD

We next asked if aggregate formation is sensitive to the intracellular concentration of the AgDD protein. We sorted HEK cells stably expressing AgDD into four groups on the basis of their S1-stabilized GFP levels: high, high-med, med-low and low (Supplementary Fig. 3b). By counting the numbers of cells with aggregates 10 min after withdrawal of S1, we observed that cells expressing higher levels of AgDD are more likely to form aggregates (high, 100%; medium-high, 95%; medium-low, 81% and low, 48%, (Supplementary Fig. 3c), suggesting that the formation of AgDD aggregates depends on the concentration of AgDD.

### Spatial control using a nuclear-localized AgDD

In order to provide spatial resolution, we next attempted to induce AgDD aggregate formation in specific cellular compartments. Protein aggregates within the nucleus are associated with neurodegenerative diseases, so we first targeted the AgDD-GFP fusion protein to the cell nucleus by fusing a nuclear localization signal to the N terminus of the AgDD construct^23^. When cells expressing nuclear AgDD are cultured in medium with S1, the GFP signal is diffusely localized in the nucleus (Fig. 3b and Supplementary Movie 5). We observed the formation of multiple, small AgDD aggregates immediately after S1 withdrawal (Fig. 3b,c; Supplementary Movies 6 and 7). These small aggregates appeared to be mobile within the cell nucleus and they coalesced somewhat but did not become one large aggregate in contrast to the behaviour of the cytosolic AgDD (Fig. 3b,c; Supplementary Movies 6 and 7). Similar results were obtained when AgDD-GFP was expressed in NIH3T3 cells (Supplementary Fig. 3d). These results demonstrate that AgDD can be used to investigate aggregate formation in specific subcellular compartments.

### AgDD induces aggregates in live animals

Finally, we used *C. elegans* to test if our AgDD system can be used to conditionally induce aggregate formation *in vivo*. We expressed AgDD in the intestinal cells of *C. elegans* using the *vha-6* promoter. Under normal growth conditions without the stabilizing S1, we did not observe AgDD signal suggesting that the AgDD fusion protein is efficiently degraded as it is when expressed in mammalian cells (Fig. 3d). When worms were grown on plates made with 50 μM S1, the stabilized AgDD-GFP is observed as a diffuse signal in the intestinal cells (Fig. 3e). When worms expressing stabilized AgDD-GFP were transferred to plates lacking S1, the AgDD started to form aggregates (Fig. 3f,g). The onset of the AgDD aggregation was slower than was observed in cultured cells, possibly because of slower rates of S1 clearance in live animals. Formation of the AgDD aggregates typically began in the anterior intestinal cells (arrow in Fig. 3f). Another interesting observation was that the larger aggregates tend to colocalize with the autofluorescence signals from gut granules (data not shown), which have been suggested to function as an aggresome-like region in intestinal cells^24^.

## Discussion

In summary, we developed a system for inducing and monitoring protein aggregates in cells using a small cell-permeable molecule. Compared with previous methods used to study aggregates, AgDD has several advantages: it has fewer off-target effects, it can be precisely regulated both spatially and temporally, and it has low toxicity for cells. Small-protein aggregates are immediately induced following withdrawal of the stabilizing ligand. Thus, this system should enable detailed mechanistic investigations of the mechanisms of the cellular response to protein aggregation. The results also demonstrate that aggregates have similar characteristics as the known filamentous aggregates from TEM and also with p62, a well-known marker for aggregates, appears to associate with the AgDD aggregates at a later stage. One possible explanation for small aggregates could be Q-bodies, which was found in yeast^25^.

Furthermore, we demonstrated that the AgDD protein can be used in living organisms, such as the nematode, *C. elegans*, and that aggregate formation could be restricted to specific cellular compartments such as the nucleus. Although further investigation is required, the behaviour of AgDD-derived aggregates in the nucleus appeared to differ from cytosolic aggregates, perhaps reflecting different protein quality-control surveillance mechanisms between these compartments^23^. This system will be extremely useful to investigate nuclear protein-quality control because nuclear protein misfolding and aggregation are strongly associated with the pathology of neurodegenerative diseases^23^. Given the high degree of control inherent in this method, the AgDD constructs may be useful in a discovery screening format to identify potential agents capable of modulating protein aggregate production.

## Methods

### Cloning

FKBP-derived AgDDs were cloned into Piggybac Transposon System pB vectors as N-terminal fusions to a SuperFolder GFP. For *C. elegans* studies, FKBP-derived AgDDs were fused to the N terminus of sfGFP, and this construct was cloned into a pSM vector where expression of the fusion protein was driven by the a *vha-6* promoter.

### Cell culture, transfections and transductions

The NIH3T3 cell lines were cultured in DMEM supplemented with 10% heat-inactivated donor bovine serum (Invitrogen), 2 mM glutamine, 100 U ml^-1^ penicillin and 100 μg ml^-1^ streptomycin. The HEK293 cell lines and U2OS cell lines were cultured with 10% heat-inactivated foetal bovine serum (Invitrogen), 2 mM glutamine, 100 U ml^-1^ penicillin and 100 μg ml^-1^ streptomycin.

To establish AgDD-expressing cell lines, cells were plated at 10 × 10^4 ^cells per well of a 6-well plate a day before transfection. The cells were co-transfected with plasmids of a pB vector and PiggyBac transposase vector following standard protocols using TransIT-LT1 (Mirus). After the cells were cultured in growth media for about a week to allow stable integration, the transfected cells were sorted by flow cytometry for high-GFP level cells.

### Withdrawal of Shield-1

To remove the Shield-1 (S1) ligand from media containing Shield-1, we directly added purified FKBP(F36V) protein (350–500 μM stock) in cultured media to achieve a final cellular protein concentration of 3.5–5 μM. The presence of extracellular FKBP creates a strong thermodynamic sink facilitating the removal of S1 from stabilizing the intracellular AgDD.

### Flow cytometry

Cells were trypsinized and resuspended in culture media before undergoing FACS sorting at the Stanford Shared FACS Facility using BD Aria II. For analytical flow cytometry, cells were trypsinized and resuspended in culture media before analysis using a BD FACS Calibur with no less than 10,000 events represented. Data were analyzed using FlowJo.

### Live-cell imaging

Time-lapse imaging was performed with a Nikon Eclipse Ti fluorescence microscope using μManager (https://www.micro-manager.org/). Cells were prepared on an 8-well Nunc Lab-Tek Chambered Coverglass (Thermo Scientific) coated with poly-Lysine (Invitrogen). During time-lapse experiments, the same conditions were maintained including a temperature of 37 °C and a CO_2_ concentration of 5%. Other live-cell images were captured on a Zeiss Axioskop 2 epifluorescence microscope equipped with a QICAM FAST 1,394 digital CCD camera.

### Immunofluorescence

Following fixation in PBS with 4% paraformaldehyde (Ted Pella) for 20 min, cells were permeabilized using PBS with 0.2% Triton-X (Sigma) for 15 min and incubated in 3% BSA (Sigma-Aldrich) in PBS for 60 min. p62 antibody (mouse, used at 1:400, BD Biosciences 610,832) incubation was carried out at 4 °C overnight followed by Alexa Fluor-conjugated secondary antibodies for 1 h at room temperature (1:1,000; Life Technologies) and Hoechst 33,342 (1:10,000; Life Technologies). Images were acquired on a custom-assembled spinning disk confocal system built around an inverted Zeiss Axiovert 200 M microscope with three lasers (442, 514 and 593.5 nm) and a CoolSNAP CCD camera from Photometrics/Roper Scientific. Imaging was carried out on an integrated ImageXpress (Molecular Devices) high-content analysis system.

### *C. elegan*s studies

*C. elegans* strain was maintained as previously described^26^. Transgenic strains were generated by injecting Pvha-6::AgDD-GFP and Podr-1::RFP plasmids into Bristol N2 strain by standard injection methods^27^. Images of fluorescently tagged fusion proteins were captured in live *C. elegans* using a Zeiss LSM710 confocal microscope (Carl Zeiss). Worms were immobilized on a 2% agarose pad with 10 μM levamisole (Sigma-Aldrich). Images were analyzed with Zen software (Carl Zeiss).

### Bioinformatic analysis

All statistical analyses were performed in R (www.rproject.org) or Prism 6. All cell image analyses were done using MATLAB and imageJ (http://imagej.nih.gov/ij/).

### Quantification of aggregates

The Haar Wavelet transform was applied once to extract the high-frequency image, and this image was resized to its original size with bi-cubic interpolation. It was then subtracted from the original image, which represents the low frequency part of the image. Preliminarily, the illumination of the image was corrected using the similar approach with seven levels of decomposition. These implementations remove noise but retain the aggregates with various size. To identify puncta of various intensity, 2D Gaussian filter was applied to the transformed image, and then if a pixel in the transformed image has 20% larger value than the corresponding pixel in the Gaussian-smoothed image, it was classified as aggregates. This binarized image was further masked by a background region, which was simply determined by a threshold of a constant value.

The average aggregates area and a number of aggregates over time were extracted from the binarized image, and then the mean and standard deviations were calculated from five different fields of views. If a number of aggregates was <100 in one field of view, the average area was set to 0 in order to suppress an error from segmentation, meaning no aggregates were observed in the image.

### Data availability

The data that support the findings of this study are available from the corresponding author upon request.

## Additional information

**How to cite this article:** Miyazaki, Y. *et al*. A method to rapidly create protein aggregates in living cells. *Nat. Commun.* 7:11689 doi: 10.1038/ncomms11689 (2016).

## Supplementary Material

Supplementary Information Supplementary Figures 1 - 3

Peer Review File

Supplementary Movie 1 Time-lapse images of representative HEK cells stably expressing AgDD. Cells cultured in media with S1 throughout the experiment.

Supplementary Movie 2 Time-lapse images of representative HEK cells stably expressing AgDD. Cells cultured in media with S1 withdrawn at 0 h.

Supplementary Movie 3 Early time-lapse images of representative HEK cells stably expressing AgDD following S1 withdrawal.

Supplementary Movie 4 Time-lapse images of representative HEK cells stably expressing AgDD. Cells cultured in media with S1 withdrawn from 0-1 h then readministered from 1-8 h.

Supplementary Movie 5 Time-lapse images of representative HEK293 cells stably expressing Nuclear AgDD. Cells cultured in media with S1 throughout the experiment.

Supplementary Movie 6 Time-lapse images of representative HEK293 cells stably expressing Nuclear AgDD. Cells cultured in media with S1 withdrawn at 0 h.

Supplementary Movie 7 Early time-lapse images of representative HEK293 cells stably expressing Nuclear AgDD following S1 removal.
