# Supplementary material for: A method to rapidly create protein aggregates in living cells
Source: Nat Commun. 2016 May 27;7:11689. doi: 10.1038/ncomms11689 (PMC4894968; doi:10.1038/ncomms11689)
Supplement: Supplementary Information — Supplementary Figures 1 - 3 [file ncomms11689-s1.pdf]

Supplementary Figure 1

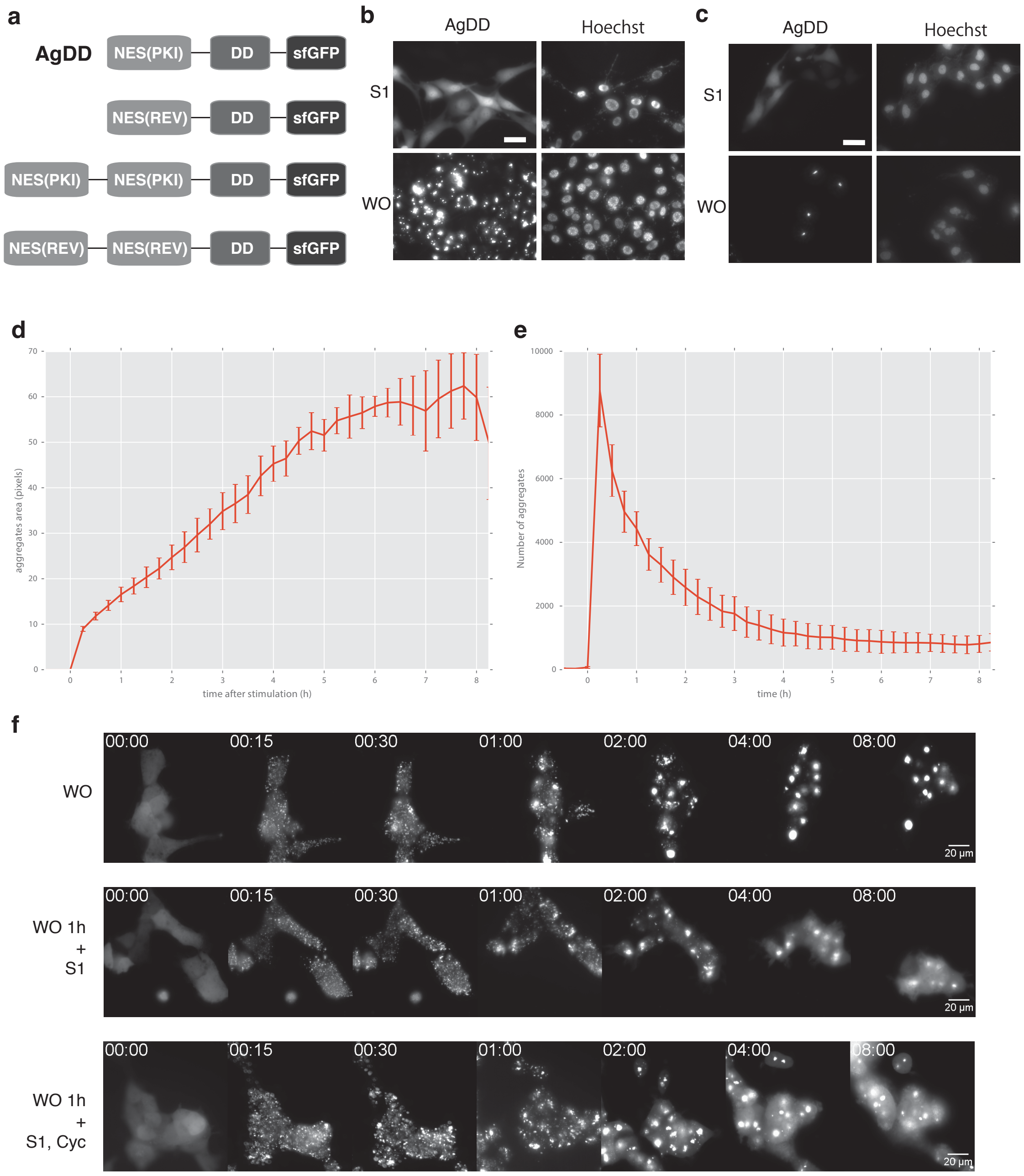

Supplementary Figure 1. Further characteristics of AgDD in cells

(a) Variation of tested NES fused DD-GFP proteins. The amino acid sequences of NES(PKI) and NES(REV) are LALKLAGLDI and LQLPPLERLTLD respectively. **(b)** Images of representative NIH 3T3 cells stably expressing AgDD before and after S1 removal for 150 min. Scale bar represents 10 μm. **(c)** Images of representative U2OS cells stably expressing AgDD before and after S1 removal for 150 min. Scale bar represent 10 μm. **(d)** Quantification of the area of single aggregates following S1 withdrawal for the indicated times. Time-lapse images of representative HEK cells stably expressing AgDD (S1 withdrawn at 0 h) were used to quantify. Error bar is STD. **(e)** Quantification of the number of aggregates following S1 withdrawal for the indicated times. Time-lapse images of representative HEK cells stably expressing AgDD (S1 withdrawn at 0 h) were used to quantify. Error bar is STD. **(f)** Time-lapse images of representative HEK cells stably expressing AgDD. Top: cells cultured in media with S1 withdrawn at 0 h; middle: cells cultured in media with S1 withdrawn from 0-1 h then readministered from 1-8 h; bottom: cells cultured in media with S1 withdrawn from 0-1 h then readministered from 1-8 h with cycloheximide. Scale bars represent 20 μm.

Supplementary Figure 2

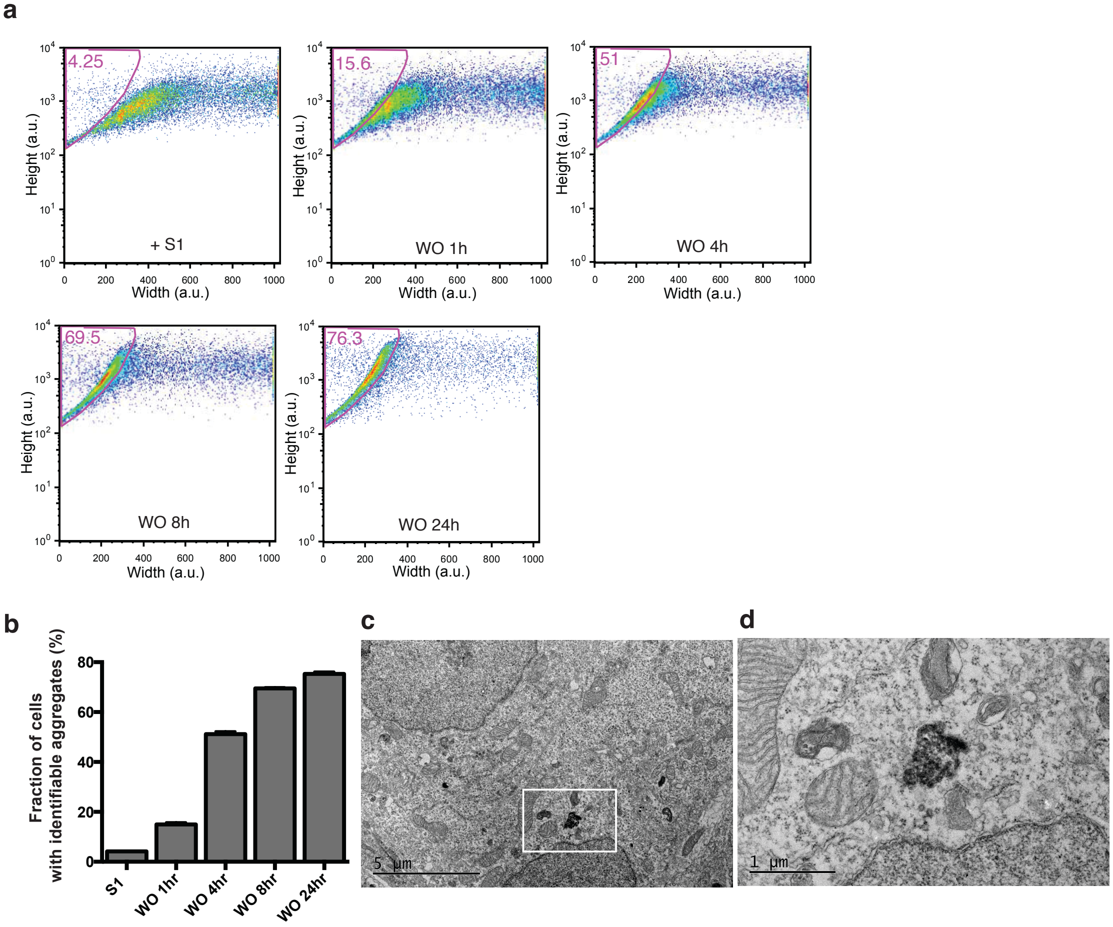

Supplementary Figure 2. AgDD aggregates monitored by PulSA method
(a) Flow cytometry analysis showing GFP signal pulse width versus height of HEK cells stably expressing AgDD before and after drug removal. Cells with aggregates are defined as the fraction of the total population within the purple gate. **(b)** Quantification of cells with aggregates by flow cytometry following S1 withdrawal for the indicated times. Replicates of n=3. Error bar is STD. **(c)** TEM images of representative HEK cells stably expressing AgDD after S1 washout for 60 min. Scale bar represents 5 μm. **(d)** Magnified TEM images of white box inset in figure S2c. Scale bar represents 1 μm.

Supplementary Figure 3

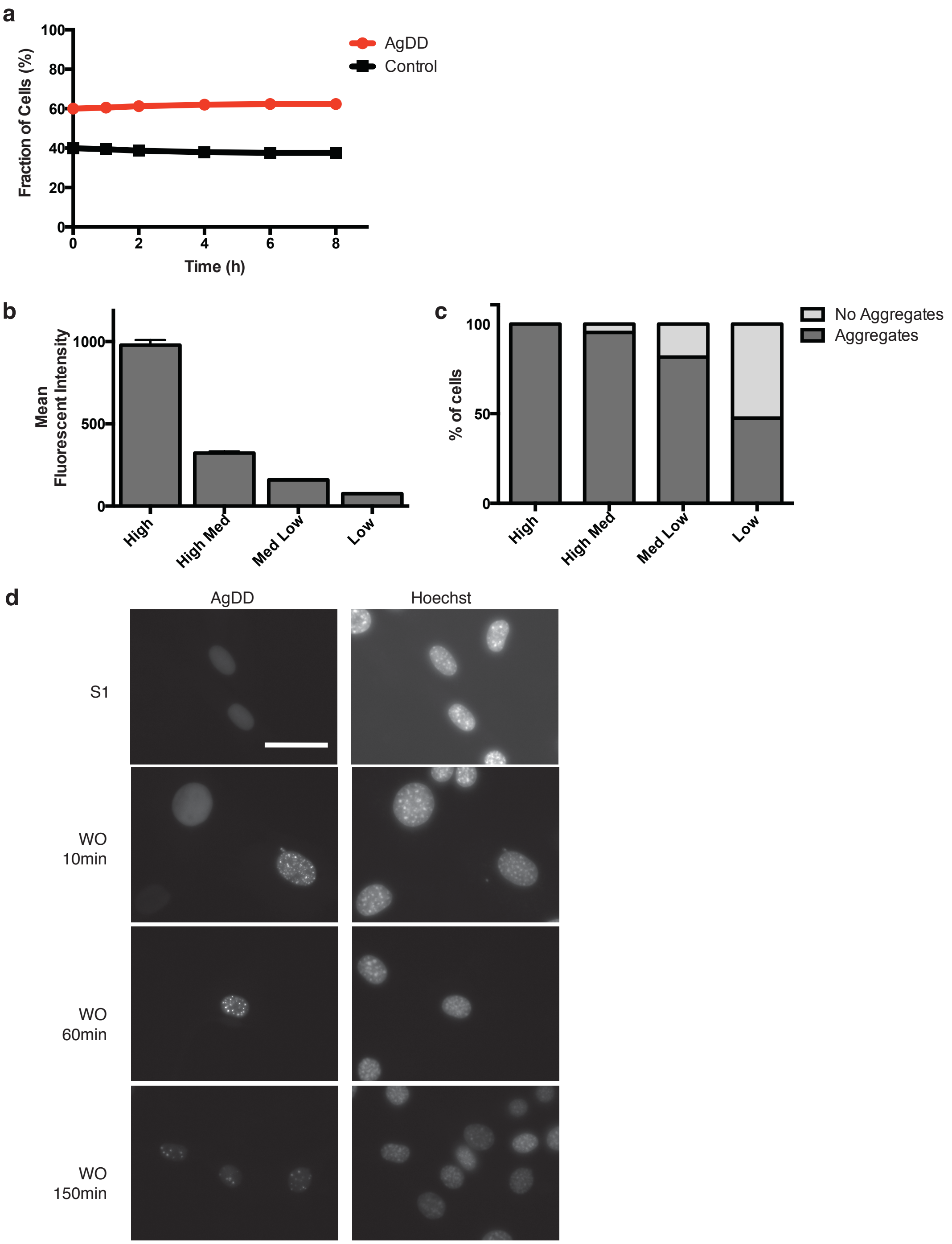

Supplementary Figure 3. Aggregates formation depends on the amount of AgDD
(a) HEK293 cells expressing AgDD and HEK293 cells expressing mCherry were mixed and co-cultured in media containing S1. The S1 was withdrawn for the indicated times, and cell populations were quantified using flow cytometry. Replicates of n=3. Error bar is STD. **(b)** Quantifying the level of AgDD expressed in four populations of HEK cells by flow cytometry. Replicates of n=3. Error bar is STD. **(c)** Using the four populations of cells in panel (b), the fraction of cells in each population with observed aggregates 10 min following S1 withdrawal. Microscopy images of HEK cells stably expressing AgDD were analyzed. **(d)** Images of representative NIH3T3 cells stably expressing Nuclear AgDD. Hoechst 33342 is used for nuclear staining. Scale bar represents 10 μm.
