# Supplementary material for: A method to rapidly create protein aggregates in living cells
Source: Nat Commun. 2016 May 27;7:11689. doi: 10.1038/ncomms11689 (PMC4894968; doi:10.1038/ncomms11689)
Supplement: Peer Review File [file ncomms11689-s2.pdf]

Editorial Note: this manuscript has been previously reviewed at another journal that is not operating a transparent peer review scheme. It was accepted without further rounds of review at Nature Communications.
